# Reappraisal inventiveness: impact of appropriate brain activation during efforts to generate alternative appraisals on the perception of chronic stress in women[Fn FN0000]

**DOI:** 10.1080/10615806.2017.1419205

**Published:** 2018-01-16

**Authors:** Corinna M. Perchtold, Andreas Fink, Christian Rominger, Hannelore Weber, Vera Loureiro de Assunção, Günter Schulter, Elisabeth M. Weiss, Ilona Papousek

**Affiliations:** ^a^ Department of Psychology, University of Graz, Graz, Austria; ^b^ Department of Psychology, University of Greifswald, Greifswald, Germany

**Keywords:** Cognitive reappraisal, emotion regulation, chronic stress, EEG asymmetry, prefrontal cortex

## Abstract

**Background and objectives**: Previous research indicated that more left-lateralized prefrontal activation during cognitive reappraisal efforts was linked to a greater capacity for generating reappraisals, which is a prerequisite for the effective implementation of cognitive reappraisal in everyday life. The present study examined whether the supposedly appropriate brain activation is relevant in terms of more distal outcomes, i.e., chronic stress perception.

**Design and methods**: Prefrontal EEG alpha asymmetry was recorded while female participants were generating reappraisals for stressful events and was correlated with their self-reported chronic stress levels in everyday life (*n* = 80).

**Results**: Women showing less left-lateralized brain activity in the ventrolateral prefrontal cortex during cognitive reappraisal efforts reported experiencing more stress in their daily lives. This effect was independent of self-efficacy beliefs in managing negative emotions.

**Conclusion**: These findings underline the practical relevance of individual differences in appropriate brain activation during emotion regulation efforts and the assumedly related basic capacity for the generation of cognitive reappraisals to the feeling of being stressed. Implications include the selection of interventions for the improvement of coping with stress in women in whom the capability for appropriate brain activation during reappraisal efforts may be impaired, e.g., due to depression or old age.

## Introduction

Stress is an inevitable part of daily life, yet if chronically enhanced, it can promote the development of affective disorders (Hammen, 2005; Hankin et al., 2015; McKlveen et al., 2016). Importantly, it is the appraisal of a stressful event rather than the event itself that figures prominently in the emotional stress response (Ellsworth & Scherer, 2003; Lazarus, 1993). As such, it is learning how to successfully reappraise adverse life events that may serve as a protective factor for severe chronic stress (Woud, Postma, Holmes, & Mackintosh, 2013). Involved in emotion regulation as well as stress coping, cognitive reappraisal refers to the process of re-interpreting the subjective meaning of an emotionally evocative event and thereby changing its emotional impact (Gross & John, 2003; Lazarus & Alfert, 1964; Lazarus & Folkman, 1984). Cognitive reappraisal is considered particularly powerful in successfully coping with stressful circumstances (Augustine & Hemenover, 2009; Webb, Miles, & Sheeran, 2012).

Adding to the valuable research on the habitual or typical use of cognitive reappraisal (see, e.g., Cutuli, 2014 for review), recent research focused on reappraisal ability in the psychometric sense, that is, to what degree people are theoretically capable of implementing cognitive reappraisal (psychometric concept of maximum performance; Cronbach, 1970). This more fundamental capacity for cognitive reappraisal was investigated in terms of an individual’s inventiveness in generating alternative appraisals of stressful situations, which can be regarded as a prerequisite for the effective implementation of cognitive reappraisal in everyday life (Weber, Assunção, Martin, Westmeyer, & Geisler, 2014). Continuing this strand of research, the present study was intended to fill a certain gap in the literature: Several studies supported a link between habitual tendencies to use cognitive reappraisal more often and better stress coping (e.g., Moore, Zoellner, & Mollenholt, 2008; Myers et al., 2012; Troy, Wilhelm, Shallcross, & Mauss, 2010). Many studies examined which brain structures are activated during reappraisal of negative stimuli (e.g., Buhle et al., 2014; Hallam et al., 2015). But there has been a relative lack of research addressing the neural equipment that allows individuals to use cognitive reappraisal effectively, with respective repercussions for how they feel in their daily lives. This research question is relevant, because this basic equipment of an individual will determine whether promoting attempts to use cognitive reappraisal more often, e.g., in cognitive behavioral therapy, will be effective at all. In individuals in whom it is impaired on account of declines in relevant brain functions, for instance, depressed (Dillon & Pizzagalli, 2013; Johnstone, van Reekum, Urry, Kalin, & Davidson, 2007; Townsend et al., 2013) older people (Opitz, Rauch, Terry, & Urry, 2012), or individuals who are neurologically impaired for other reasons, training of other strategies such as distraction could then be more effective (Scult, Knodt, Swartz, Brigidi, & Hariri, 2017; Smoski, LaBar, & Steffens, 2014).

Recent experimental findings identified neural correlates of the inventiveness in generating alternative appraisals of stressful situations: More left-lateralized activation in the lateral prefrontal cortex during reappraisal efforts (assessed by EEG alpha asymmetry), specifically in the ventral region, was linked to a greater number and diversity of generated reappraisals (Papousek et al., 2017). The location was in line with several brain imaging studies which had indicated the involvement of lateral prefrontal cortex, particularly in the left hemisphere, in the generation of reappraisals (Dillon & Pizzagalli, 2013; Dörfel et al., 2014; Kalisch, 2009; Ochsner, Silvers, & Buhle, 2012; Phan et al., 2005; Price, Paul, Schneider, & Siegle, 2013; for meta-analysis, see Buhle et al., 2014). These findings, by implication, suggested that the proneness or capability to recruit the supposedly appropriate brain circuits when faced with the demand of reappraising a stressful event (mirrored in left-lateralized prefrontal activation) is also beneficial in terms of more distal outcomes such as individuals’ chronic stress experience (Papousek et al., 2017). However, this has not yet been empirically demonstrated. Accordingly, the purpose of this study was to examine if the pattern of activation that was shown to be related to greater reappraisal inventiveness in previous research (Papousek et al., 2017) is relevant in terms of chronic stress perception.

We studied this issue in the context of laterality research, which focuses on relative differences of activation in the left and right cortical hemispheres and has proved to be relevant in this particular context. According to the capability model of frontal EEG alpha asymmetry (Coan, Allen, & McKnight, 2006), the individual’s capability or typical mode to adapt to the specific demands of a certain situation is reflected in the individual’s recruitment of appropriate brain circuits in that situation, which produces characteristic asymmetry changes in the brain. These asymmetry changes, recorded in a respective context, index the individual’s capability to effectively process the specific demands and, consequently, may be indicative of traits related to psychological health and well-being (see also Allen & Reznik, 2015; Beeney, Levy, Gatzke-Kopp, & Hallquist, 2014; Cole, Zapp, Nelson, & Perez-Edgar, 2012; Goodman, Rietschel, Lo, Costanzo, & Hatfield, 2013; Liu, Sarapas, & Shankman, 2016; Papousek, Reiser, et al., 2013, 2014; Pérez-Edgar, Kujawa, Nelson, Cole, & Zapp, 2013; Stewart, Coan, Towers, & Allen, 2014). More generally, several studies indicated functional deficits when brain circuits that are associated with these functions were inadequately activated, and that lateralized activation of specific relevant brain regions was linked to better performance on associated tasks (Davidson, Chapman, Chapman, & Henriques, 1990; Gur et al., 1994, 2000; Gur & Reivich, 1980; Papousek, Murhammer, & Schulter, 2011; Papousek & Schulter, 2004; Wendt & Risberg, 1994). The capability model represents one of several important advancements of prefrontal brain asymmetry research that in part may be hampered by too simplistic approaches such as the reliance on resting data (see Miller, Crocker, Spielberg, Infantolino, & Heller, 2013 for a critical review).

While not specifically concerned with the recruitment of relevant brain regions during efforts to cognitively reappraise stressful events, the findings of several studies in the tradition of EEG alpha asymmetry research suggested importance of relative left-sided prefrontal activation for coping with stressful events (Blackhart & Kline, 2005; Goodman et al., 2013; Jackson et al., 2003; Lopez-Duran, Nusslock, George, & Kovacs, 2012; Papousek et al., 2014). The hypothesized link is additionally suggested by an fMRI study showing cognitive reappraisal efforts to elicit left-lateralized ventrolateral prefrontal activation in healthy individuals, whereas depressed patients displayed bilateral prefrontal activation (Johnstone et al., 2007). The authors interpreted the absence of clear left-lateralized activation as inefficient or inappropriate engagement of prefrontal regulatory circuitry, which may relate to the difficulties of depressed patients to adequately cope with adverse events (e.g., Beauregard, Paquette, & Levesque, 2006). Likewise, a lack of left-frontal activation during reappraisal efforts was found in elderly people, which concomitantly diminished their reappraisal success in terms of decreasing negative affect (Opitz et al., 2012). In accordance with the capability model of frontal EEG asymmetry, in the present study EEG alpha asymmetry was recorded while participants were generating cognitive reappraisals in the Reappraisal Inventiveness Test (RIT; Weber et al., 2014). While typical cognitive reappraisal tasks make it difficult to ascertain that participants are actually compliant when asked to use this specific strategy (mostly while watching negative affective material), and a large proportion of participants seem to actually not adhere to specific emotion regulation instructions (Demaree, Robinson, Pu, & Allen, 2006), using the RIT allowed to objectively monitor participants’ compliance with reappraisal instructions. In particular, the RIT involves the active generation of many different reappraisals of anger-eliciting scenarios, depicting a conflictual relationship with another person who wilfully or carelessly induces harm. Anger regulation constitutes a relevant context, because higher levels of anger experience are related to perceptions of higher stress levels (e.g., Diong et al., 2005; Johnson, Galambos, & Krahn, 2014).

In order to exclude that potential correlations between individual differences in brain activation during efforts to generate reappraisals and perceptions of chronic stress are due to efficacy beliefs as the decisive factor, we also assessed the participants’ self-efficacy beliefs in managing negative emotions. Self-efficacy beliefs in managing emotional experiences refer to a personality trait that may affect the individual’s appraisal of his or her circumstances and responses to stressful events and, hence, may strongly influence self-reported chronic stress levels (Petrides, 2011; Petrides, Pita, & Kokkinaki, 2007). Reciprocal influences between cognitive-emotional abilities such as reappraisal inventiveness and perceived efficacy in managing emotions seem likely. Self-efficacy beliefs are supposed to affect to what degree individuals make efforts to actively cope with stressful events (i.e., make use of their abilities), and better abilities should at least to some extent predict more perceived efficacy in managing one’s emotions (Bandura, 2001; Gohm, Corser, & Dalsky, 2005). There is evidence for substantial correlations between perceived efficacy in managing emotions and several indexes of well-being (Caprara & Steca, 2005; Freudenthaler, Neubauer, Gabler, Scherl, & Rindermann, 2008; Lightsey et al., 2012; Milioni et al., 2015; Palesh et al., 2006).

We hypothesized that individuals showing brain activation patterns during efforts to generate cognitive reappraisals that were suggested to be more appropriate (efficient) by previous research (i.e., more relative left-lateralized ventrolateral prefrontal activation; Papousek et al., 2017) will report less chronic stress experience in everyday life. In line with the EEG asymmetry research tradition, we infer increased cortical activation from relative decreases of alpha power (see Allen, Coan, & Nazarian, 2004 for a review of evidence and Harmon-Jones, 2006; Michels et al., 2010; Scheeringa et al., 2011 for recent experimental research supporting the assumption that EEG alpha band activity obtained in time frames of several seconds or minutes is inversely related to cortical activity). Furthermore, we expected at least some of the shared variance between brain activation during reappraisal efforts and chronic stress experience to be independent of perceived efficacy in managing emotions, thereby corroborating the importance of the brain’s basic capability to generate reappraisals for perceptions of chronic stress. Finally, it was tested whether chronic stress perception may also be correlated with overt performance differences on the used experimental cognitive reappraisal task, taking different types of cognitive reappraisal into account.

## Methods

### Participants

Eighty participants aged between 18 and 35 years (*M* = 22.7, SD = 3.5) completed the experiment with all required data. All participants were university students enrolled in various fields and female. A student sample was chosen, because the contents of the used material (RIT; Weber et al., 2014) had been tailored for a student population. A female-only sample was tested in order to avoid any confounding effects produced by potential gender differences in emotion-related abilities and habits (e.g., Domes et al., 2010; Freudenthaler & Papousek, 2013). Accordingly, women may be more motivated to downregulate anger for social reasons (Evers, Fischer, Rodriguez Mosquera, & Manstead, 2005) and reported using both adaptive and maladaptive emotion regulation strategies more frequently than men (Nolen-Hoeksema & Aldao, 2011). The sample size was based on a priori power analysis considering effect sizes observed in previous relevant research, common retest-correlations among repeated measures of EEG variables, and commonly recommended type 1 error probability (*α* = .05) and power (1 – *β* = 0.80; Bühner & Ziegler, 2009). Women who reported having a neuropsychiatric disease or using psychoactive medication were not included in the study. Seven out of the 80 participants were smokers. Right-handedness was confirmed using a standardized hand skill test (Papousek & Schulter, 1999; Steingrüber & Lienert, 1971). Participants were requested to refrain from alcohol for 12 hours and from coffee and other stimulating beverages for two hours prior to their lab appointment, and to come to the session well rested. The study was performed in accordance with the American Psychological Association’s Ethics Code and the Declaration of Helsinki and was approved by the local ethics committee. Participants gave their written consent to participate in the study.

### Reappraisal task

The four items of the RIT (Weber et al., 2014) were used to establish situations in which the participants were required to generate alternative appraisals of stressful events. Four additional items were added that matched the main characteristics of the original ones (Papousek et al., 2017). The items consist of anger-eliciting vignettes that are supplemented by a matching photograph to make the situation more vivid. Each vignette was presented on a computer screen for 20 s. Participants were instructed to imagine the situation happening to them and to generate as many different ways as possible to think about or appraise the situation in a way that diminishes anger. They were instructed to press a button whenever a new appraisal came to mind and to vocalize the idea concisely in one or two short sentences immediately after pressing the button. Then they were asked to press the button again, and the task was resumed until the allotted time of 3 min had elapsed. In doing so, EEG segments related to the production of reappraisals could be separated from segments contaminated with the production of speech. This protocol has proved to be eminently suitable in previous relevant research (Fink, Benedek, Grabner, Staudt, & Neubauer, 2007; Papousek et al., 2017). Participants’ vocal responses were audiotaped for later analysis, and adherence to the protocol was carefully monitored.

### EEG recording and quantification

EEG was recorded from 19 channels according to the international 10–20 system, using a Brainvision BrainAmp Research Amplifier (Brain Products) and a stretchable electrode cap, referenced to the nose and re-referenced offline to a mathematically averaged ears reference (Hagemann, 2004). Impedance was kept below 5 kΩ for all electrodes. EOG measures were obtained for identification of ocular artifacts. All data were inspected visually, in order to eliminate intervals in which ocular or muscle artifacts occurred. For the assessment of EEG asymmetry during the reappraisal task, only the time frames in which participants were mentally generating alternative appraisals were used, that is, reading and speaking intervals were excluded. To preclude potential influences of finger movements on frontal EEG asymmetry (e.g., Harmon-Jones, 2006; Peterson, Shackman, & Harmon-Jones, 2008), time frames were cut with a distance of 450 ms to each button press (cf. Shibasaki & Hallett, 2006). Power spectra (epoch length 1 s, overlapping 50%, Hanning window) were averaged across all artifact-free intervals for an individual. Following the common approach in the field, power within the alpha frequency band (8–12 Hz) was used for the analyses. Laterality coefficients (LC) were computed for each electrode pair as LC = ((*R* − *L*)/(*R* + *L*)) × 100, where *R* denotes the electrode over the right hemisphere and *L* denotes the homologous electrode over the left hemisphere. This asymmetry ratio is equivalent to another common metric (ln *R* – ln *L*), with which it is virtually perfectly correlated (Davidson, 1988; Papousek & Schulter, 2002). However, LC allows easier comparison of data from different studies, different frequency bands, and locations (Pivik et al., 1993), and has been used in numerous EEG studies in relevant research contexts (e.g., Papousek et al., 2011, 2014; Papousek, Reiser, et al., 2013; Papousek, Reiser, Weber, Freudenthaler, & Schulter, 2012; Papousek & Schulter, 2004; Papousek, Schulter, et al., 2013). Positive values of LC indicate relatively greater left than right hemisphere cortical activity (inverse of alpha).

### Reappraisal inventiveness

Participants’ responses to the RIT items were used for the assessment of behavioral measures of their reappraisal inventiveness. Following the scoring procedure of the RIT (Weber et al., 2014), RIT-fluency was calculated as the total number of generated non-identical reappraisals (Cronbach’s *α* = .93). Reappraisals were categorized according to the category scheme of the RIT (Weber et al., 2014): generating positive aspects (positive re-interpretation); problem-oriented (casting the situation in terms of how the induced harm could be reduced); de-emphasizing the negative impact of the harm induced and/or the instigator’s wrong-doing; revenge (casting the situation in terms of getting even). Responses were independently rated by two experimenters. Inter-rater reliabilities with two-way random, single measure ICC (95% confidence intervals, consistency) were = .93 for RIT-fluency, and ICC = .87, ICC = .92, ICC = .95, and ICC = .93 for the total number of responses categorized as positive re-interpretation, problem-oriented, de-emphasizing, and revenge, respectively.

### Self-report measures

*Chronic stress experience*. The short form (screening scale) of the Trier Inventory for Chronic Stress (TICS; Schulz, Schlotz, & Becker, 2004) was used for the assessment of participants’ perceptions of chronic stress during the last three months (12 items rated on 5-point Likert scales ranging from 0 (never) to 4 (very often), *α* = .89). Scores ranged from 0 to 36 (*M* = 16.9, SD = 8.7).

*Depression*. The Center for Epidemiologic Studies depression scale (CES-D, German version, Hautzinger & Bailer, 1993) is composed of 20 items, rated from 0 (rarely or none of the time – less than 1 day) to 4 (most or all of the time – 5 to 7 days; *α* = .82). It refers to mood and attributions over the past week and is designed for measuring sub-clinical depressive experiences in the general population (Wood, Taylor, & Joseph, 2010). Scores ranged from 1 to 31 (*M* = 11.7, SD = 6.6).

*Perceived efficacy in managing negative emotions*. The emotion regulation subscale of the Self-report Emotional Ability Scale (SEAS; Freudenthaler & Neubauer, 2005) was used, which assesses how able individuals feel to regulate negative affect in their everyday life. It includes six items (rated on 6-point Likert scales; *α* = .75).

### Procedure

Participants completed the handedness test and were seated in an acoustically and electrically shielded examination room. After electrodes were attached, participants were instructed to relax and sat quietly with closed eyes for two minutes to adapt. Subsequently, the EEG was recorded during an initial two minutes resting condition (open eyes, participants were instructed to rest their eyes on a filled green circle on the screen). Then, the participants were instructed for the task and were given a practice item. After completing the task (while the EEG was again recorded) electrodes were detached, and the participants were given the opportunity to wash and dry their hair. Finally, they completed the questionnaires.

### Statistical analysis

Three linear regressions were calculated using resting EEG alpha asymmetry at an F7/F8, Fp1/Fp2, or F3/F4 to predict asymmetry during the generation of reappraisals at the respective electrode site. The residualized scores of prefrontal alpha asymmetry during the generation of reappraisals were used in the main analysis. This was done to ensure that the analyzed variability was due to the activation during the generation of reappraisals, and not to individual differences in baseline levels at that electrode site (i.e., to general inter-individual differences irrespective of the demands at hand; cf. Papousek et al., 2014). The main research question was examined with one standard multiple regression analysis using prefrontal alpha asymmetry during the generation of reappraisals at the three prefrontal electrode sites (F7/F8, Fp1/Fp2, and F3/F4) and perceived efficacy in managing negative emotions (SEAS) as predictors, and chronic stress (TICS) as the dependent variable. An analogous analysis was done with depression (CES-D) as the dependent variable. These results can be found in the supplemental material.

In order to test for the possibility that perceived efficacy in managing negative emotions mediates the relationship between prefrontal asymmetry and chronic stress, an additional mediation analysis was performed using PROCESS (Hayes, 2013). PROCESS is based on the regression-based path-analytic framework and estimates the indirect effect and bias-corrected confidence intervals. An indirect effect is considered significant when the confidence intervals do not include zero. The level of this significance was assessed using Sobel tests. All analyses were based on 5000 bootstrapping samples (Hayes, 2013).

Additionally, a multiple regression analysis was run to test whether more appropriate (i.e., more left-lateralized) prefrontal activity during efforts to generate reappraisals predicts higher performance on the task (cf. Papousek et al., 2017). Finally, it was also tested in analogous multiple regression analyses, whether perceptions of stress may be predicted by the behavioral indexes of reappraisal inventiveness.

## Results

### Brain activation during the generation of alternative appraisals

EEG alpha asymmetry during efforts to generate cognitive reappraisals at the ventral electrodes (F7/F8) predicted participants’ perceptions of chronic stress in daily life (*sr* = −.24, *p* = .006; *F*(4,75) = 16.2, *p* < .001). Women showing less left-lateralized activity at that site while generating reappraisals reported greater levels of chronically experienced stress. This relationship was independent of the participants’ efficacy beliefs in managing negative emotions, which were correlated with perceptions of chronic stress on their part (*r* = −.63, *p* < .001). No relationship was observed between chronic stress and EEG alpha asymmetry during reappraisal generation at the most rostral (Fp1/Fp2; *sr* = −.04, *p* = .660) and the dorsal electrode positions (F3/F4; *sr* = −.15, *p* = .073). See Table 1 for a summary of the results of the main analysis. When perceived efficacy in managing negative emotions is omitted from the regression, the relevant correlation between asymmetry at the ventral electrodes and chronic stress remains unchanged (with efficacy beliefs included *sr* = −.24; with efficacy beliefs excluded *sr* = −.23, *p* = .045).

**Table 1.** Prediction of perception of chronic stress by prefrontal EEG alpha asymmetries during efforts to generate cognitive reappraisals.

EEG alpha asymmetry at prefrontal electrode sites	*r* (*p*)	*sr* (*p*)
F7/F8 (ventral)	−.23 (.042)	−.24 (.006)
Fp1/Fp2 (most rostral)	.07 (.534)	.04 (.660)
F3/F4 (dorsal)	−.09 (.439)	.15 (.073)
Efficacy beliefs	−.63 (<.001)	−.64 (<.001)

Note*: r* = zero-order correlation, *sr* = semipartial correlation, *p* = *p*-value. Negative values of EEG alpha asymmetry denote higher alpha activity in the left than in the right hemisphere, that is, relatively lower left than right hemisphere cortical activity. Residualized scores adjusted for the asymmetry at the respective electrode site in resting conditions.

There was no significant indirect (mediation) effect of frontal EEG alpha asymmetry through self-efficacy beliefs on chronic stress experience (F7/F8; *b* = .23, SE = .546; Fp1/Fp2; *b* = −.13, SE = .608; F3/F4; *b* = .59, SE = .610). See Table 2 for all regression mediation results.

**Table 2.** Outcomes of mediation analyses from prefrontal EEG alpha asymmetry to chronic stress perception assessing indirect effects of perceived self-efficacy in dealing with negative emotions.

EEG alpha asymmetry Self-efficacy Chronic stress	*B*					Bootstrap results for ab (95% CI)		Bootstrap results for ab sizes (95% CI)		
	*R*^2^	*c*′ (*p*)	*a* (*p*)	*b* (*p*)	*ab* (*p*)	Lower	Upper	*k*^2^	Lower	Upper
F7/F8 (ventral) Self-efficacy Chronic stress	.44	1.77 (.021)	−.21 (.710)	−1.08 (<.001)	.23 (.712)	−.8875	1.3331	.0114	−.0345	.0894
Fp1/Fp2 (most rostral) Self-efficacy Chronic stress	.41	.75 (.333)	.12 (.835)	−1.09 (<.001)	−.13 (.836)	−1.4289	1.0138	−.0023	−.0330	.0162
F3/F4 (dorsal) Self-efficacy Chronic stress	.40	.17 (.823)	−.55 (.342)	−1.09 (<.001)	.59 (.348)	−.5344	1.9110	.0073	−.0102	.0744

Note: *B* = unstandardized regression weight, *c’* = direct effect of predictor on outcome while controlling for the mediator, *a *= effect of the predictor on the mediator, *b* = effect of the mediator on the outcome, *ab* = indirect effect of predictor on outcome through the mediator, *R* ^2^ **= amount of variance explained by the model, *CI* = confidence intervals; *k* ^2^ = effect size in kappa squared, *p* = *p*-value.

### Behavioral measures of reappraisal inventiveness

A lower number of generated cognitive reappraisals (RIT-fluency) were associated with less left-lateralized activation at the ventral and most rostral electrodes during efforts to generate alternative appraisals (F7/F8; *sr* = .20, *p* = .055; *F*(3,74) = 3.76, *p* < .001; Fp1/Fp2; *sr* = .25, *p *= .026; F3/F4; *sr* = −.25, *p* = .022).

Levels of perceived chronic stress were not significantly predicted by the total number of generated cognitive reappraisals (RIT-fluency, *sr* = −.10, *p* = .247; *F*(2,77) = 26.7, *p* < .001). Looking closer into the categories of alternative appraisals, the number of responses categorized as positive re-interpretation showed a significant negative correlation with perceived chronic stress levels when efficacy beliefs in managing of emotions held constant (*sr* = −.18, *p* = .038; *F*(2,77) = 29.3, *p* < .001; zero-order correlation *r* = −.14, *p* = .209). No correlations were observed for responses categorized as problem-oriented (*sr* = −.02, *p* = .809; *F*(2,77) = 25.7, *p* < .001), de-emphasizing (*sr* = −.02, *p* = .821; *F*(2,77) = 25.7, *p* < .001), and revenge (*sr* = −.05, *p* = .579; *F*(2,77) = 25.9, *p* < .001). On average, *M* = 5.1 (SD = 3.9) responses were classified as positive re-interpretation, *M* = 20.9 (SD = 9.9) as problem-oriented, *M* = 4.7 (SD = 4.9) as de-emphasizing, and *M* = 3.5 (SD = 3.7) as and revenge.

## Discussion

In the present study, women with higher chronic stress experience showed less left-lateralized brain activity in the ventrolateral prefrontal cortex during cognitive reappraisal efforts. Previous results in this research area had revealed that this activation pattern was associated with reduced inventiveness to generate suitable reappraisals for self-relevant, stressful situations (Papousek et al., 2017), a finding that was confirmed in the present study. Together, these findings nicely fit the notion that a more strongly left-lateralized activation in the ventrolateral prefrontal cortex during reappraisal efforts is more appropriate (efficient in terms of emotion regulation).

Correspondingly, in depressed as well as in elderly people showing reduced reappraisal success, attenuated activation in the left hemisphere or less left-lateralized activation was found in ventrolateral regions during cognitive reappraisal efforts (fMRI; Johnstone et al., 2007; Opitz et al., 2012). A recent review more generally indicated reduced recruitment of the ventrolateral prefrontal cortex during efforts to downregulate negative emotion in psychiatric disorders (Zilverstand, Parvaz, & Goldstein, 2017). The importance of left ventrolateral prefrontal activation is further corroborated by a number of imaging studies consistently showing increased activation in the left ventrolateral prefrontal region during instructed reappraisal, particularly at earlier periods of the experimental reappraisal phases that were presumably dominated by efforts to generate alternative appraisals (other activations also occur, which probably are related to other processes; Dillon & Pizzagalli, 2013; Kalisch, 2009; Ochsner et al., 2012; Phan et al., 2005). EEG alpha asymmetry studies, too, showed left-lateralized activation in the ventrolateral prefrontal region during efforts to generate cognitive reappraisals (Choi, Sekiya, Minote, & Watanuki, 2016; Papousek et al., 2017). This convergence across methods supports the basic assumption that relative decreases of EEG alpha power in one hemisphere indicate relative greater activation of that hemisphere, which has been questioned and may not hold in all instances (Miller et al., 2013). Importantly, cognitive reappraisal was shown to specifically recruit left ventrolateral prefrontal cortex when compared with other strategies such as expressive suppression or distraction (fMRI; Dörfel et al., 2014; Price et al., 2013). Note that while these findings converge in that they indicate the importance of left-lateralized activity in the ventrolateral prefrontal region, neither those findings nor the present results exclude that lateralized activity in other brain areas are also important or may even be more important for that matter.

The present findings also demonstrated that the effect of individual differences in brain activation during reappraisal efforts on chronic stress experience is also present when adjusting for individual differences in perceived efficacy in managing negative emotions. This corroborates the importance of the brain’s basic capability to generate reappraisals for perceptions of chronic stress. The results suggested that both are important independently of each other: the brain’s basic capability to generate alternative appraisals as well as the confidence that one’s emotion regulation efforts are effective. This is in line with the suggestion that feelings of efficacy in managing negative emotions may be a necessary precondition to cope successfully with stressful events, because they make sure that individuals attempt to cope actively with stressful events, irrespectively of their abilities (Gohm et al., 2005). The formal size of the correlations might suggest that self-efficacy may play a much greater role than the basic foundation provided by the brain. However, the relatively high correlation between self-efficacy and chronic stress experience should not be over-estimated in this case, because it may partly be attributed to facet duplication or common method assessment (Freudenthaler & Papousek, 2013). Consequently, although confidence in one’s own emotion regulation efforts is crucial in successfully dealing with negative emotions, one needs to account for the brain’s basic capability to generate reappraisals when trying to understand practical ramifications of various emotion regulation strategies.

To the attentive reader, it might occur that the obtained individual differences in alpha asymmetry during the reappraisal task might have, in part, originated from the right-hand button presses shortly before participants vocalized their reappraisal ideas. There is evidence that repeated unilateral hand contractions may cause contralateral motor cortex activation to spread to prefrontal, particularly dorsolateral prefrontal cortex areas (F3/F4; Harmon-Jones, 2006; Peterson, Gravens, & Harmon-Jones, 2011; Peterson et al., 2008). However, the influence of motor preparation in tiny self-initiated finger movements such as single button presses on frontal asymmetry is less clear (e.g., Miller & Tomarken, 2001). Nonetheless, we had reduced the likelihood of possible influences of motor preparatory activity to a minimum by excluding the 450 ms preceding each button press from the EEG data analysis (cf. late Bereitschaftspotential; Shibasaki & Hallett, 2006). That as well as the fact that motor preparation predominantly influences activity at more posterior electrode sites rules out that participants’ motor responses may have had decisively affected our results.

The importance of the individual’s inventiveness in generating cognitive reappraisals is further substantiated by the correlation of lower chronic stress perception with higher scores on the behavioral test in terms of a higher number of generated positive re-interpretations. It is important to note that reappraisal inventiveness in the RIT (Weber et al., 2014) refers to an ability measure in a narrower sense, as used in psychometrics (Cronbach, 1970), capturing what people can do at their best, thus reflecting the fundamental capacity for generating reappraisals rather than their typical behavior or typical achievement. While widely used in psychology (e.g., intelligence tests) as well as in neurology (e.g., motor performance tests) and psychiatry (e.g., neuropsychological testing), the use of maximum performance measures is largely novel to the field of cognitive reappraisal.

Certainly, this basic (brain) capacity for implementing cognitive reappraisals only covers a certain aspect of an individual’s ability to effectively use cognitive reappraisal for negative affect regulation. At first glance, in daily life it might seem to be of even greater importance to produce one high-quality reappraisal than a pool of different appraisals to effectively mitigate the emotional impact of stressful situations. However, the ability to generate a variety of potential alternative appraisals for a given situation is one vital factor in the successful use of cognitive reappraisal. Individuals may rely on one or a few typical strategies for reappraisal that have become habitual over time and are sufficient until they are confronted with new situations, in which they cannot rely on their routine strategies. For effective emotion regulation, a broad repertoire of possible reappraisals and their flexible, situation-appropriate use is necessary. Thus, the effectiveness of reappraisal efforts depends in part on the pool of appraisals generated (see also Wisco & Nolen-Hoeksema, 2010). The capability to generate a broad pool of appraisals increases the individual’s potential to select one that is most effective for successful coping with the specific stressful situation at hand.

In this study, there was a specific negative correlation between perceived chronic stress and the number of alternative appraisals categorized as positive re-interpretation. This suggests that, in addition to the general capacity to generate cognitive reappraisals, it may be the quality of the generated ideas that is critically related to emotional well-being. Research on the impact of different types of cognitive reappraisal has been sparse to date. The category of positive re-interpretation refers to situation-focused reappraisal, which aims at re-interpreting the nature of the emotional events themselves, thereby changing their meaning; as opposed to self-focused reappraisal, which involves altering the personal relevance of events (Ochsner et al., 2004). Experimental findings suggested that situation-focused reappraisal may be a more effective emotion regulation strategy than self-focused reappraisal, at least as reducing immediate negative responses to unpleasant stimuli is concerned (Willroth & Hilimire, 2016). Practicing reappraisal in terms of changing the meaning of a stimulus had more beneficial effects on later emotion regulation than practicing cognitive detachment strategies (Schartau, Dalgleish, & Dunn, 2009). In line with these empirical findings, cognitive reappraisal as used in psychotherapy typically focuses on re-interpreting the meaning of a stimulus (Dunn, Billotti, Murphy, & Dalgleish, 2009).

Interestingly, in two studies that instructed participants to use positive re-interpretation/situation-focused reappraisal, these strategies recruited a network comprising (left) lateral prefrontal cortex, while other brain regions were activated during attempts to reduce the personal relevance of emotional stimuli (Dörfel et al., 2014; Falquez et al., 2014). However, a meta-analysis of studies using various types of instructed reappraisal did not yield a clear picture on that matter (Webb et al., 2012), most likely because the analyzed studies had not specifically focused on differences between types of reappraisal, and thus no particular measures were taken to ensure that participants adhered to specific instructions. Again, it is important to note that it was the capability for positive re-interpretation that was negatively related to chronic stress experience in the present study, but not the self-reported typical use of this strategy, for which different relations might be found (e.g., Denny & Ochsner, 2014).

An important limitation of the present study is its correlational/cross-sectional design, which limits causal interpretations of the relations. While this study’s research background suggests appropriate brain activation during cognitive reappraisal efforts being the cause and the perception of chronic stress the effect, reverse influences might also play a role. Effective implementation of cognitive reappraisal probably depends on the functionality of executive functions such as cognitive switching, memory updating, and the inhibition of dominant responses (Joormann & Gotlib, 2010; Malooly, Genet, & Siemer, 2013; Pe, Raes, Kuppens, & di Pellegrino, 2013; Weber et al., 2014), which are linked to the recruitment of left prefrontal activation on their part (e.g., Badre & Wagner, 2007; Hirshorn & Thompson-Schill, 2006; Jahanshahi, Dirnberger, Fuller, & Frith, 2000; Jonides & Nee, 2006). There is some evidence that stress may impair core executive functions such as cognitive flexibility and cognitive (but not response) inhibition (Shields, Sazma, & Yonelinas, 2016). In line with this, animal research suggested that chronic stress increases synaptic inhibition onto prefrontal glutamatergic output neurons, thereby impairing the influence of the prefrontal cortex in controlling stress reactivity (McKlveen et al., 2016). It was suggested that these processes may foster attention to highly salient information (Vogel, Fernandez, Joels, & Schwabe, 2016), which may be beneficial for efficient threat responding, but may counteract the implementation of cognitive reappraisal, thus potentially initiating a vicious cycle.

Secondly, this study only included female participants. While a women-only sample was chosen to avoid confounding effects of sex differences in cognitive emotion regulation, this approach limits the generalizability of the findings. Further research is warranted to look into potential sex differences with respect to generating cognitive reappraisals for anger-eliciting events, both psychometrically and at the level of the brain. Another limitation of the present study is that the items in the cognitive reappraisal task concerned anger-evoking events only. Being a special case of negatively valenced and approach-oriented emotional states, more angry states have been linked to more left-lateralized activation in the prefrontal region (Harmon-Jones, 2004; Harmon-Jones, Gable, & Peterson, 2010; Stewart, Levin-Silton, Sass, Heller, & Miller, 2008). Hence, one might wonder whether the findings could perhaps be explained by individual differences in the experience of anger. However, if individual differences in anger experience had decisively influenced the results, they would indicate that greater experience/poorer regulation of anger was linked to less chronic stress perception. As poor regulation of anger is prospectively associated with higher perceptions of chronic stress and depression (Chue, Gunthert, Ahrens, & Skalina, 2017; Johnson et al., 2014; Naragon-Gainey & Watson, 2014), this seems very unlikely. Moreover, effects of anger on prefrontal EEG alpha asymmetry were typically observed at dorsal electrode sites (F3/F4; Harmon-Jones et al., 2010). Still, replication of the present findings with vignettes inducing emotional states other than anger is certainly required. Lastly, addressing the specificity of our findings, it must be emphasized that some specificity was demonstrated on the level of the brain, showing unique contributions of left-lateralized activity at the ventrolateral (but not other frontal) electrode positions during the generation of anger-reducing cognitive reappraisals in predicting chronic stress experience. Further research may expand the specificity issue by also including more posterior sites. On the behavioral level, more research will be required to identify which processes and aspects of well-being are most affected by the investigated brain process. There certainly may be considerable overlapping between chronic stress experience and other affective traits and disturbances.

Taken together, the present study demonstrated that women differ in their brain’s proneness to recruit appropriate brain activation while attempting to generate cognitive reappraisals of stressful situations. These inter-individual differences seem to have sustainable effects on psychological well-being. The findings further suggest that if the brain’s basic capability to generate alternative appraisals is impaired, reinforcing individuals’ efforts to re-interpret stressful events alone may not suffice to impede or reverse stress-related psychopathological developments.

## References

[CIT0001] Allen J. J. B., Coan J. A., & Nazarian M. (2004). Issues and assumptions on the road from raw signals to metrics of frontal EEG asymmetry in emotion. *Biological Psychology*, , 183–218. doi: 10.1016/j.biopsycho.2004.03.007 15130531

[CIT0002] Allen J. J. B., & Reznik S. J. (2015). Frontal EEG asymmetry as a promising marker of depression vulnerability: Summary and methodological considerations. *Current Opinion in Psychology*, , 93–97. doi: 10.1016/j.copsyc.2014.12.017 26462291 PMC4599354

[CIT0003] Augustine A. A., & Hemenover S. H. (2009). On the relative effectiveness of affect regulation strategies: A meta-analysis. *Cognition and Emotion*, , 1181–1220. doi: 10.1080/02699930802396556

[CIT0004] Badre D., & Wagner A. D. (2007). Left ventrolateral prefrontal cortex and the cognitive control of memory. *Neuropsychologia*, , 2883–2901. doi: 10.1016/j.neuropsychologia.2007.06.015 17675110

[CIT0005] Bandura A. (2001). Social cognitive theory: An agentic perspective. *Annual Review of Psychology*, , 1–26. doi:10.1111/1467-839x.00024 doi: 10.1146/annurev.psych.52.1.1 11148297

[CIT0006] Beauregard M., Paquette V., & Levesque J. (2006). Dysfunction in the neural circuitry of emotional self-regulation in major depressive disorder. *Neuroreport*, , 843–846. doi: 10.1097/01.wnr.0000220132.32091.9f 16708026

[CIT0007] Beeney J. E., Levy K. N., Gatzke-Kopp L. M., & Hallquist M. N. (2014). EEG asymmetry in borderline personality disorder and depression following rejection. *Personality Disorders*, , 178–185. doi: 10.1037/per0000032 24364503 PMC4137775

[CIT0008] Blackhart G. C., & Kline J. P. (2005). Individual differences in anterior EEG asymmetry between high and low defensive individuals during a rumination/distraction task. *Personality and Individual Differences*, , 427–437. doi: 10.1016/j.paid.2005.01.027

[CIT0009] Buhle J. T., Silvers J. A., Wager T. D., Lopez R., Onyemekwu C., Kober H., … , Ochsner K. N. (2014). Cognitive reappraisal of emotion: A meta-analysis of human neuroimaging studies. *Cerebral Cortex*, , 2981–2990. doi: 10.1093/cercor/bht154 23765157 PMC4193464

[CIT0010] Bühner M., & Ziegler M. (2009). *Statistik für Psychologen und Sozialwissenschafter*. München: Pearson.

[CIT0011] Caprara G. V., & Steca P. (2005). Affective and social self-regulatory efficacy beliefs as determinants of positive thinking and happiness. *European Psychologist*, , 275–286. doi: 10.1027/1016-9040.10.4.275 16584101

[CIT0012] Choi D., Sekiya T., Minote N., & Watanuki S. (2016). Relative left frontal activity in reappraisal and suppression of negative emotion: Evidence from front alpha asymmetry. *International Journal of Psychophysiology*, , 37–44. doi:10.1016/j.ipsycho.2016.09.018 doi: 10.1016/j.ijpsycho.2016.09.018 27693504

[CIT0013] Chue A. E., Gunthert K. C., Ahrens A. H., & Skalina L. M. (2017). How does social anger expression predict later depression symptoms? It depends on how often one is angry. *Emotion*, , 6–10. doi: 10.1037/emo0000239 27819444

[CIT0014] Coan J. A., Allen J. J. B., & McKnight P. E. (2006). A capability model of individual differences in frontal EEG asymmetry. *Biological Psychology*, , 198–207. doi: 10.1016/j.biopsycho.2005.10.003 16316717 PMC2835626

[CIT0015] Cole C., Zapp D. J., Nelson K., & Perez-Edgar K. (2012). Speech presentation cues moderate frontal EEG asymmetry in socially withdrawn young adults. *Brain and Cognition*, , 156–162. doi: 10.1016/j.bandc.2011.10.013 22169714 PMC3268053

[CIT0016] Cronbach L. J. (1970). *Essentials of psychological testing* (3rd ed.). New York: Harper.

[CIT0017] Cutuli D. (2014). Cognitive reappraisal and expressive suppression strategies role in the emotion regulation: An overview on their modulatory effects and neural correlates. *Frontiers in Systems Neuroscience*, , 175–181. doi: 10.3389/fnsys.2014.00175 25285072 PMC4168764

[CIT0018] Davidson R. J. (1988). EEG measures of cerebral asymmetry: Conceptual and methodological issues. *International Journal of Neuroscience*, , 71–89. doi: 10.3109/00207458808985694 3290140

[CIT0019] Davidson R. J., Chapman J. P., Chapman L. J., & Henriques J. B. (1990). Asymmetrical brain electrical activity discriminates between psychometrically-matched verbal and spatial cognitive tasks. *Psychophysiology*, , 528–543. doi: 10.1111/j.1469-8986.1990.tb01970.x 2274616

[CIT0020] Demaree H. A., Robinson J. L., Pu J., & Allen J. J. B. (2006). Strategies actually employed during response-focused emotion regulation research: Affective and physiological consequences. *Cognition Emotion*, , 1248–1260. doi: 10.1080/02699930500405303

[CIT0021] Denny B. T., & Ochsner K. (2014). Behavioural effects of longitudinal training in cognitive reappraisal. *Emotion*, , 425–433. doi: 10.1037/a0035276 24364856 PMC4096123

[CIT0022] Dillon D. G., & Pizzagalli D. A. (2013). Evidence of successful modulation of brain activation and subjective experience during reappraisal of negative emotion in unmedicated depression. *Psychiatry Research: Neuroimaging*, , 99–107. doi: 10.1016/j.pscychresns.2013.01.001 PMC3640775 23570916

[CIT0023] Diong S. M., Bishop G. D., Enkelmann H. C., Tong E. M., Why Y. P., Ang J. C., & Khader M. (2005). Anger, stress, social support and health: Modelling the relation-ships. *Psychology and Health*, , 467–495. doi: 10.1080/0887044040512331333960

[CIT0024] Domes G., Schulze L., Böttger M., Grossmann A., Hauenstein K., Wirtz P. H., … Herpertz S. C. (2010). The neural correlates of sex differences in emotional reactivity and emotion regulation. *Human Brain Mapping*, , 758–769. doi: 10.1002/hbm.20903 19957268 PMC6871188

[CIT0025] Dörfel D., Lamke J.-P., Hummel F., Wagner U., Erk S., & Walter H. (2014). Common and differential neural networks of emotion regulation by detachment, reinterpretation, distraction, and expressive suppression: A comparative fMRI investigation. *NeuroImage*, , 298–309. doi: 10.1016/j.neuroimage.2014.06.051 24993897

[CIT0026] Dunn B. D., Billotti D., Murphy V., & Dalgleish T. (2009). The consequences of effortful emotion regulation when processing distressing material: A comparison of suppression and acceptance. *Behaviour Research and Therapy*, , 761–773. doi: 10.1016/j.brat.2009.05.007 19559401 PMC2764381

[CIT0027] Ellsworth P. C., & Scherer K. R. (2003). Appraisal processes in emotion. In Davidson R. J., Scherer K. R., & Goldsmith H. H. (Eds.), *Handbook of affective sciences* (pp. 572–595). New York: Oxford University Press.

[CIT0028] Evers C., Fischer A. H., Rodriguez Mosquera P. M., & Manstead A. S. R. (2005). Anger and social appraisal: A “spicy” sex difference? *Emotion*, , 258–266. doi: 10.1037/1528-3542.5.3.258 16187862

[CIT0029] Falquez R., Couto B., Ibanez A., Freitag M. T., Berger M., Arens E. A. … Barnow S. (2014). Detaching from the negative by reappraisal: The role of right superior frontal gyrus (BA9/32). *Frontiers in Behavioral Neuroscience*, , 165–181. doi: 10.3389/fnbeh.2014.00165 24847230 PMC4023069

[CIT0030] Fink A., Benedek M., Grabner R. H., Staudt B., & Neubauer A. C. (2007). Creativity meets neuroscience: Experimental tasks for the neuroscientific study of creative thinking. *Methods*, , 68–76. doi: 10.1016/j.ymeth.2006.12.001 17434417

[CIT0031] Freudenthaler H. H., & Neubauer A. J. (2005). Emotional intelligence: The convergent and discriminant validities of intra- and interpersonal emotional abilities. *Personality and Individual Differences*, , 569–579. doi: 10.1016/j.paid.2005.02.004

[CIT0032] Freudenthaler H. H., Neubauer A. J., Gabler P., Scherl W. G., & Rindermann H. (2008). Testing and validating the trait emotional intelligence questionnaire (TEIQue) in a German-speaking sample. *Personality and Individual Differences*, , 673–678. doi: 10.1016/j.paid.2008.07.014

[CIT0033] Freudenthaler H. H., & Papousek I. (2013). The typical and maximum performance of intra- and interpersonal emotion management. In Mohiyeddini C. (Ed.), *Emotional relationships: Types, challenges, and physical/mental health impacts* (pp. 125–160). Hauppauge, NY: Nova Science.

[CIT0034] Gohm C. L., Corser G. C., & Dalsky D. J. (2005). Emotional intelligence under stress: Useful, unnecessary, or irrelevant. *Personality and Individual Differences*, , 1017–1028. doi: 10.1016/j.paid.2005.03.018

[CIT0035] Goodman R. N., Rietschel J. C., Lo L.-C., Costanzo M. E., & Hatfield B. D. (2013). Stress, emotion regulation and cognitive performance: The predictive contributions of trait and state relative frontal EEG alpha asymmetry. *International Journal of Psychophysiology*, , 115–123. doi: 10.1016/j.ijpsycho.2012.09.008 23022494

[CIT0036] Gross J. J., & John O. P. (2003). Individual differences in two emotion regulation processes: Implications for affect, relationships, and well-being. *Journal of Personality and Social Psychology*, , 348–362. doi: 10.1037/0022-3514.85.2.348 12916575

[CIT0037] Gur R. C., Alsop D., Glahn D., Petty R., Swanson C. L., Maldijan J. A., & Gur R. E. (2000). An fMRI study of sex differences in regional activation to a verbal and a spatial task. *Brain and Language*, , 157–170. doi: 10.1006/brln.2000.2325 10950912

[CIT0038] Gur R. C., Ragland J. D., Resnick S. M., Skolnick B. E., Jaggi J., Muenz L., & Gur R. E. (1994). Lateralized increases in cerebral blood flow during performance of verbal and spatial tasks: Relationship with performance level. *Brain and Cognition*, , 244–258. doi: 10.1006/brcg.1994.1013 8185896

[CIT0039] Gur R. C., & Reivich M. (1980). Cognitive task effects on hemispheric blood flow in humans: Evidence for individual differences in hemispheric activation. *Brain and Language*, , 78–92. doi: 10.1016/0093-934x(80)90073-5 7357384

[CIT0040] Hagemann D. (2004). Individual differences in anterior EEG asymmetry: Methodological problems and solutions. *Biological Psychology*, , 157–182. doi: 10.1016/j.biopsycho.2004.03.006 15130530

[CIT0041] Hallam G. P., Webb T. L., Sheeran P., Miles, E., Wilkinson I. D., & Hunter M. D. (2015). The neural correlates of emotion regulation by implementation intentions. *PLoS ONE*, , 1–21. doi: 10.1371/journal.pone.0119500 PMC4370584 25798822

[CIT0042] Hammen C. (2005). Stress and depression. *Annual Review of Clinical Psychology*, , 293–319. doi: 10.1146/annurev.clinpsy.1.102803.143938 17716090

[CIT0043] Hankin B. L., Young J. F., Abela J. R. Z., Smolen A., Jenness J. L., Gulley L. D., … Oppenheimer C. W. (2015). Depression from childhood into late adolescence: Influence on gender, development, genetic susceptibility, and peer stress. *Journal of Abnormal Psychology*, , 803–816. doi: 10.1037/abn0000089 26595469 PMC4662048

[CIT0044] Harmon-Jones E. (2004). Contributions from research on anger and cognitive dissonance to understanding the motivational functions of asymmetrical frontal brain activity. *Biological Psychology*, , 51–76. doi: 10.1016/j.biopsycho.2004.03.003 15130525

[CIT0045] Harmon-Jones E. (2006). Unilateral right-hand contractions cause contralateral alpha power suppression and approach motivational affective experience. *Psychophysiology*, , 598–603. doi: 10.1111/j.1469-8986.2006.00465.x 17076816

[CIT0046] Harmon-Jones E., Gable P. A., & Peterson C. K. (2010). The role of asymmetric frontal cortical activity in emotion-related phenomena: A review and update. *Biological Psychology*, , 451–462. doi: 10.1016/j.biopsycho.2009.08.010 19733618

[CIT0047] Hautzinger M., & Bailer M. (1993). *Allgemeine Depressions Skala. General depression scale*. Weinheim: Beltz. doi: 10.1026//012-1924.47.4.208

[CIT0048] Hayes A. (2013). *Introduction to mediation, moderation and conditional process analysis*. New York: Guilford Press.

[CIT0049] Hirshorn E. A., & Thompson-Schill S. L. (2006). Role of the left inferior frontal gyrus in covert word retrieval: Neural correlates of switching during verbal fluency. *Neuropsychologia*, , 2547–2557. doi: 10.1016/j.neuropsychologia.2006.03.035 16725162

[CIT0050] Jackson D. C., Mueller C. J., Dolski I., Dalton K. M., Nitschke J. B., Urry H. L., & Davidson R. J. (2003). Now you feel it, now you don’t: Frontal brain electrical asymmetry and individual differences in emotion regulation. *Psychological Science*, , 612–617. doi: 10.1046/j.0956-7976.2003.psci_1473.x 14629694

[CIT0051] Jahanshahi M., Dirnberger G., Fuller R., & Frith C. D. (2000). The role of the dorsolateral prefrontal cortex in random number generation: A study with positron emission tomography. *NeuroImage*, , 713–725. doi: 10.1006/nimg.2000.0647 11112403

[CIT0052] Johnson M. D., Galambos N. L., & Krahn H. J. (2014). Depression and anger across 25 years: Changing vulnerabilities in the VSA model. *Journal of Family Psychology*, , 225–235. doi: 10.1037/a0036087 24588603

[CIT0053] Johnstone T., van Reekum C. M., Urry H. L., Kalin N. H., & Davidson R. J. (2007). Failure to regulate: Counterproductive recruitment of top-down prefrontal-subcortical circuitry in major depression. *Journal of Neuroscience*, , 8877–8884. doi: 10.1523/jneurosci.2063-07.2007 17699669 PMC6672169

[CIT0054] Jonides J., & Nee D. E. (2006). Brain mechanisms of proactive interference in working memory. *Neuroscience*, , 181–193. doi:10 1016/j.neuroscience.2005.06.042 doi: 10.1016/j.neuroscience.2005.06.042 16337090

[CIT0055] Joormann J., & Gotlib I. H. (2010). Emotion regulation in depression: Relation to cognitive inhibition. *Cognition and Emotion*, , 281–298. doi: 10.1080/02699930903407948 20300538 PMC2839199

[CIT0056] Kalisch R. (2009). The functional neuroanatomy of reappraisal: Time matters. *Neuroscience & Biobehavioral Reviews*, , 1215–1226. doi: 10.1016/j.neubiorev.2009.06.003 19539645

[CIT0057] Lazarus R. S. (1993). From psychological stress to the emotions: A history of changing outlooks. *Annual Review of Psychology*, , 1–21. doi:10.1146/annurev.psych.44.1.1 doi: 10.1146/annurev.ps.44.020193.000245 8434890

[CIT0058] Lazarus R. S., & Alfert E. (1964). Short-circuiting of threat by experimentally altering cognitive appraisal. *Journal of Abnormal and Social Psychology*, , 195–205. doi: 10.1037/h0044635 14213291

[CIT0059] Lazarus R. S., & Folkman S. (1984). *Stress, appraisal, and coping*. New York: Springer.

[CIT0060] Lightsey O. R., McGhee R., Erin A., Gharibian Gharghani G., Rarey E. B., Daigle R. P., … Powell K. (2012). Self-efficacy for affect regulation as a predictor of future life satisfaction and moderator of the negative affect – life satisfaction relationship. *Journal of Happiness Studies*, , 1–18. doi: 10.1007/s10902-011-9312-4

[CIT0061] Liu H., Sarapas C., & Shankman S. A. (2016). Anticipatory reward deficits in melancholia. *Journal of Abnormal Psychology*, , 631–640. doi: 10.1037/abn0000172 27175986 PMC4925246

[CIT0062] Lopez-Duran N. L., Nusslock R., George C., & Kovacs M. (2012). Frontal EEG asymmetry moderates the effects of stressful life events on internalizing symptoms in children at familial risk for depression. *Psychophysiology*, , 510–521. doi: 10.1111/j.1469-8986.2011.01332.x 22220930 PMC4063310

[CIT0063] Malooly A. M., Genet J. J., & Siemer M. (2013). Individual differences in reappraisal effectiveness: The role of affective flexibility. *Emotion*, , 302–313. doi: 10.1037/a0029980 23163706

[CIT0064] McKlveen J. M., Morano R. L., Fitzgerald M., Zoubovsky S., Cassella S. N., Scheimann J. R., … Herman J. P. (2016). Chronic stress increases prefrontal inhibition: A mechanism for stress-induced prefrontal dysfunction. *Biological Psychiatry*, , 754–764. doi: 10.1016/j.biopsych.2016.03.2101 27241140 PMC5629635

[CIT0065] Michels L., Bucher K., Lüchinger R., Klaver P., Martin E., Jeanmonod D., & Brandeis D. (2010). Simultaneous EEG-fMRI during a working memory task: Modulations in low and high frequency bands. *PLoS ONE*, , 1–15. doi: 10.1371/journal.pone.0010298 PMC2858659 20421978

[CIT0066] Milioni M., Alessandri G., Eisenberg N., Castellani V., Zuffianò A., Vecchione M., & Caprara G. V. (2015). Reciprocal relations between emotional self-efficacy beliefs and ego-resiliency. *Journal of Personality*, , 552–563. doi: 10.1111/jopy.12131 25204666 PMC4709220

[CIT0067] Miller G. A., Crocker L. D., Spielberg J. M., Infantolino Z. P., & Heller W. (2013). Issues in localization of brain function: The case of lateralized frontal cortex in cognition, emotion, and psychopathology. *Frontiers in Integrative Neuroscience*, , 1–9. doi: 10.3389/fnint.2013.00002 23386814 PMC3558680

[CIT0068] Miller A., & Tomarken A. J. (2001). Task-dependent changes in frontal brain asymmetry: Effects of incentive cues, outcome expectancies, and motor responses. *Psychophysiology*, , 500–511. doi: 10.1111/1469-8986.3830500 11352139

[CIT0069] Moore S. A., Zoellner L. A., & Mollenholt N. (2008). Are expressive suppression and cognitive reappraisal associated with stress-related symptoms? *Behaviour Research and Therapy*, , 993–1000. doi: 10.1016/j.brat.2008.05.001 18687419 PMC2629793

[CIT0070] Myers S. B., Sweeney A. C., Popick V., Wesley K., Bordfeld A., & Fingerhut R. (2012). Self-care practices and perceived stress levels among psychology graduate students. *Training and Education in Professional Psychology*, , 55–66. doi: 10.1037/a0026534

[CIT0071] Naragon-Gainey K., & Watson D. (2014). Consensually defined facets of personality as prospective predictors of change in depression symptoms. *Assessment*, , 387–403. doi: 10.1177/1073191114528030 24671734

[CIT0072] Nolen-Hoeksema S., & Aldao A. (2011). Gender and age differences in emotion regulation strategies and their relationship to depressive symptoms. *Personality and Individual Differences*, , 704–708. doi: 10.1016/j.paid.2011.06.012

[CIT0073] Ochsner K. N., Ray R. D., Cooper J. C., Robertson E. R., Chopra S., Gabrieli J. D. E., & Gross J. J. (2004). For better or for worse: Neural systems supporting the cognitive down- and up-regulation of negative emotion. *NeuroImage*, , 483–499. doi: 10.1016/j.neuroimage.2004.06.030 15488398

[CIT0074] Ochsner K. N., Silvers J. A., & Buhle J. T. (2012). Functional imaging studies of emotion regulation: A synthetic review and evolving model of the cognitive control of emotion. *The Annals of the New York Academy of Science*, 1251, E1–E24. doi: 10.1111/j.1749-6632.2012.06751.x PMC4133790 23025352

[CIT0075] Opitz P. C., Rauch L. C., Terry D. P., & Urry H. L. (2012). Prefrontal mediation of age differences in cognitive reappraisal. *Neurobiology of Aging*, , 645–655. doi: 10.1016/j.neurobiolaging.2010.06.004 20674090

[CIT0076] Palesh O. G., Shaffer T., Larson B. A., Edsall S., Chen X.-H., Koopman C., … Parsons R. (2006). Emotional self-efficacy, stressful life-events, and satisfaction with social support in relation to mood disturbance among women living with breast cancer in rural communities. *The Breast Journal*, , 123–129. doi: 10.1111/j.1075-122x.2006.00219.x 16509836

[CIT0077] Papousek I., Murhammer D., & Schulter G. (2011). Intra- and interindividual differences in lateralized cognitive performance and asymmetrical EEG activity in the frontal cortex. *Brain and Cognition*, , 225–231. doi: 10.1016/j.bandc.2010.11.013 21145157

[CIT0078] Papousek I., Reiser E. M., Schulter G., Fink A., Holmes E. A., Niederstätter H., … Weiss E. M. (2013). Serotonin transporter genotype (5-HTTLPR) and electrocortical responses indicating the sensitivity to negative emotional cues. *Emotion*, , 1173–1181. doi: 10.1037/a0033997 24040881 PMC3948098

[CIT0079] Papousek I., Reiser E. M., Weber B., Freudenthaler H. H., & Schulter G. (2012). Frontal brain asymmetry and affective flexibility in an emotional contagion paradigm. *Psychophysiology*, , 489–498. doi: 10.1111/j.1469-8986.2011.01324.x 22176666

[CIT0080] Papousek I., & Schulter G. (1999). Quantitative assessment of five behavioural laterality measures: Distribution of scores and intercorrelations among right-handers. *Laterality*, , 345–362. doi: 10.1080/713754344 15513122

[CIT0081] Papousek I., & Schulter G. (2002). Covariations of EEG asymmetries and emotional states indicate that activity at frontopolar locations is particularly affected by state factors. *Psychophysiology*, , 350–360. doi: 10.1017/S0048577201393083 12212654

[CIT0082] Papousek I., & Schulter G. (2004). Manipulation of frontal brain asymmetry by cognitive tasks. *Brain and Cognition*, , 43–51. doi: 10.1016/S0278-2626(03)00258-6 14733899

[CIT0083] Papousek I., Schulter G., Weiss E. M., Samson A. C., Freudenthaler H. H., & Lackner H. K. (2013). Frontal brain asymmetry and transient cardiovascular responses to the perception of humor. *Biological Psychology*, , 114–121. doi: 10.1016/j.biopsycho.2012.12.004 23274171

[CIT0084] Papousek I., Weiss E. M., Perchtold C. M., Weber H., Assuncao V. L., Schulter G., … Fink A. (2017). The capacity for generating cognitive reappraisals is reflected in asymmetric activation of frontal brain regions. *Brain Imaging and Behavior*, , 577–590. doi: 10.1007/s11682-016-9537-2 26935554 PMC5408052

[CIT0085] Papousek I., Weiss E. M., Schulter G., Fink A., Reiser E. M., & Lackner H. K. (2014). Prefrontal EEG alpha asymmetry changes while observing disaster happening to other people: Cardiac correlates and prediction of emotional impact. *Biological Psychology*, , 184–194. doi: 10.1016/j.biopsycho.2014.09.001 25224180

[CIT0086] Pe M. L., Raes F., Kuppens P., & di Pellegrino G. (2013). The cognitive building blocks of emotion regulation: Ability to update working memory moderates the efficacy of rumination and reappraisal on emotion. *PLoS ONE*, , e69071. doi: 10.1371/journal.pone.0069071 23874872 PMC3715480

[CIT0087] Pérez-Edgar K., Kujawa A., Nelson K., Cole C., & Zapp D. J. (2013). The relation between electroencephalogram asymmetry and attention biases to threat at baseline and under stress. *Brain and Cognition*, , 337–343. doi: 10.1016/j.bandc.2013.05.009 PMC3722560 23807238

[CIT0088] Peterson C. K., Gravens L. C., & Harmon-Jones E. (2011). Asymmetric frontal cortical activity and negative affective responses to ostracism. *Social Cognitive and Affective Neuroscience*, , 277–285. doi: 10.1093/scan/nsq027 PMC3110424 20360350

[CIT0089] Peterson C. K., Shackman A. J., & Harmon-Jones E. (2008). The role of asymmetrical frontal cortical activity in aggression. *Psychophysiology*, , 86–92. doi: 10.1111/j.1469-8986.2007.00597.x 17850239

[CIT0090] Petrides K. V. (2011). Ability and trait emotional intelligence. In Chamorro-Premuzic T., von Stumm S., & Furnham A. (Eds.), *The Blackwell-Wiley handbook of individual differences* (pp. 656–678). New York: Wiley.

[CIT0091] Petrides K. V., Pita R., & Kokkinaki F. (2007). The location of trait emotional intelligence in personality factor space. *British Journal of Psychology*, , 273–289. doi: 10.1348/000712606X120618 17456273

[CIT0092] Phan K. L., Fitzgerald D. A., Nathan P. J., Moore G. J., Uhde T. W., & Tancer M. E. (2005). Neural substrates for voluntary suppression of negative affect: A functional magnetic resonance imaging study. *Biological Psychiatry*, , 210–219. doi: 10.1016/j.biopsych.2004.10.030 15691521

[CIT0093] Pivik R. T., Broughton R. J., Coppola R., Davidson R. J., Fox N., & Nuwer M. R. (1993). Guidelines for the recording and quantitative analysis of electroencephalographic activity in research contexts. *Psychophysiology*, , 547–558. doi: 10.1111/j.1469-8986.1993.tb02081.x 8248447

[CIT0094] Price R. B., Paul B., Schneider W., & Siegle G. J. (2013). Neural correlates of three neurocognitive intervention strategies: A preliminary step towards personalized treatment for psychological disorders. *Cognitive Therapy Research*, , 657–672. doi: 10.1007/s10608-012-9508-x 23935231 PMC3736858

[CIT0095] Schartau P. E. S., Dalgleish T., & Dunn B. D. (2009). Seeing the bigger picture: Training in perspective broadening reduces self-reported affect and psychophysiological response to distressing films and autobiographical memories. *Journal of Abnormal Psychology*, , 15–27. doi: 10.1037/a0012906 19222310

[CIT0096] Scheeringa R., Fries P., Petersson K. M., Oostenveld R., Grothe I., Norris D. G., … Bastiaansen M. C. (2011). Neuronal dynamics underlying high- and low-frequency EEG oscillations contribute independently to the human BOLD signal. *Neuron*, , 572–583. doi: 10.1016/j.neuron.2010.11.044 21315266

[CIT0097] Schulz P., Schlotz W., & Becker P. (2004). *Trierer Inventar zum chronischen* [*Stress: TICS* [*Trier inventory for chronic stress*]]. Göttingen: Hogrefe.

[CIT0098] Scult M. A., Knodt A. R., Swartz J. R., Brigidi B. D., & Hariri A. R. (2017). Thinking and feeling: Individual differences in habitual emotion regulation and stress-related mood are associated with prefrontal executive control. *Clinical Psychological Science*, , 150–157. doi: 10.1177/2167702616654688 28191365 PMC5298870

[CIT0099] Shibasaki H., & Hallett M. (2006). What is the Bereitschaftspotential? *Clinical Neurophysiology*, , 2341–2356. doi: 10.1016/j.clinph.2006.04.025 16876476

[CIT0100] Shields G. S., Sazma M. A., & Yonelinas A. P. (2016). The effects of acute stress on core executive functions: A meta-analysis and comparison with cortisol. *Neuroscience and Biobehavioral Reviews*, , 651–668. doi: 10.1016/j.neubiorev.2016.06.038 27371161 PMC5003767

[CIT0101] Smoski M. J., LaBar K. S., & Steffens D. C. (2014). Relative effectiveness of reappraisal and distraction in regulating emotion in late-life depression. *The American Journal of Geriatric Psychiatry*, , 898–907. doi: 10.1016/j.jagp.2013.01.070 24021222 PMC3949222

[CIT0102] Steingrüber H., & Lienert G. (1971). *Hand-Dominanz-Test*. Göttingen: Hogrefe.

[CIT0103] Stewart J. L., Coan J. A., Towers D. N., & Allen J. J. B. (2014). Resting and task-elicited prefrontal EEG alpha asymmetry in depression: Support for the capability model. *Psychophysiology*, , 446–455. doi: 10.1111/psyp.12191 24611480 PMC3984363

[CIT0104] Stewart J. L., Levin-Silton R., Sass S. M., Heller W., & Miller G. (2008). Anger style, psychopathology, and regional brain activity. *Emotion*, , 701–713. doi: 10.1037/a0013447 18837620 PMC3047003

[CIT0105] Townsend J. D., Torrisi S. J., Lieberman M. D., Sugar C. A., Bookheimer S. Y., & Altshuler L. L. (2013). Frontal-amygdala connectivity alterations during emotion downregulation in bipolar I disorder. *Biological Psychiatry*, , 127–135. doi: 10.1016/j.biopsych.2012.06.030 22858151 PMC3525751

[CIT0106] Troy A. S., Wilhelm F. H., Shallcross A. J., & Mauss I. B. (2010). Seeing the silver lining: Cognitive reappraisal ability moderates the relationship between stress and depressive symptoms. *Emotion*, , 783–795. doi: 10.1037/a0020262 21058843 PMC3278301

[CIT0107] Vogel A., Fernandez G., Joels M., & Schwabe L. (2016). Cognitive adaptation under stress: A case for the mineralocorticoid receptor. *Trends in Cognitive Science*, , 192–203. doi: 10.1016/j.tics.2015.12.003 26803208

[CIT0108] Webb T. L., Miles E., & Sheeran P. (2012). Dealing with feeling: A meta-analysis of the effectiveness of strategies derived from the process model of emotion regulation. *Psychological Bulletin*, , 775–808. doi: 10.1037/a0027600 22582737

[CIT0109] Weber H., Assunção V. L., Martin C., Westmeyer H., & Geisler F. C. (2014). Reappraisal inventiveness: The ability to generate different reappraisals of critical situations. *Cognition and Emotion*, , 345–360. doi: 10.1080/02699931.2013.832152 24044510

[CIT0110] Wendt P. E., & Risberg J. (1994). Cortical activation during visual spatial processing: Relation between hemispheric asymmetry of blood flow and performance. *Brain and Cognition*, , 87–103. doi: 10.1006/brcg.1994.1005 8123265

[CIT0111] Willroth E. C., & Hilimire M. R. (2016). Differential effects of self- and situation-focused reappraisal. *Emotion*, , 468–474. doi: 10.1037/emo0000139 26641270

[CIT0112] Wisco B., & Nolen-Hoeksema S. (2010). Interpretation bias and depressive symptoms: The role of self-relevance. *Behaviour Research and Therapy*, , 1113–1122. doi: 10.1016/j.brat.2010.08.004 20810100

[CIT0113] Wood A. M., Taylor P. J., & Joseph S. (2010). Does the CES-D measure a continuum from depression to happiness? Comparing substantive and artifactual models. *Psychiatry Research*, , 120–123. doi: 10.1016/j.psychres.2010.02.003 20207424

[CIT0114] Woud M. L., Postma P., Holmes E. A., & Mackintosh B. (2013). Reducing analogue trauma symptoms by computerized reappraisal training – considering a cognitive prophylaxis? *Journal of Behavioral Therapy and Experimental Psychiatry*, , 312–315. doi:10.1016/jbtep.2013.01.003 doi: 10.1016/j.jbtep.2013.01.003 PMC3617361 23454552

[CIT0115] Zilverstand A., Parvaz M. A., & Goldstein R. Z. (2017). Neuroimaging cognitive reappraisal in clinical populations to define neural targets for enhancing emotion regulation. A systematic review. *NeuroImage*, , 105–116. doi: 10.1016/j.neuroimage.2016.06.009 PMC5145785 27288319

